# Differential mirror neuron system (MNS) activation during action observation with and without social-emotional components in autism: a meta-analysis of neuroimaging studies

**DOI:** 10.1186/s13229-020-00374-x

**Published:** 2020-09-29

**Authors:** Melody M. Y. Chan, Yvonne M. Y. Han

**Affiliations:** Department of Rehabilitation Sciences, The Hong Kong Polytechnic University, 11 Yuk Choi Road, Hung Hom, Kowloon, Hong Kong

**Keywords:** Meta-analysis, Autism, Action observation, Mirror neuron, Emotion, fMRI, ES-SDM

## Abstract

**Background:**

Impaired imitation has been found to be an important factor contributing to social communication deficits in individuals with autism spectrum disorder (ASD). It has been hypothesized that the neural correlate of imitation, the mirror neuron system (MNS), is dysfunctional in ASD, resulting in imitation impairment as one of the key behavioral manifestations in ASD. Previous MNS studies produced inconsistent results, leaving the debate of whether “broken” mirror neurons in ASD are unresolved.

**Methods:**

This meta-analysis aimed to explore the differences in MNS activation patterns between typically developing (TD) and ASD individuals when they observe biological motions with or without social-emotional components. Effect size signed differential mapping (ES-SDM) was adopted to synthesize the available fMRI data.

**Results:**

ES-SDM analysis revealed hyperactivation in the right inferior frontal gyrus and left supplementary motor area in ASD during observation of biological motions. Subgroup analysis of experiments involving the observation of stimuli with or without emotional component revealed hyperactivation in the left inferior parietal lobule and left supplementary motor during action observation without emotional components, whereas hyperactivation of the right inferior frontal gyrus was found during action observation with emotional components in ASD. Subgroup analyses of age showed hyperactivation of the bilateral inferior frontal gyrus in ASD adolescents, while hyperactivation in the right inferior frontal gyrus was noted in ASD adults. Meta-regression within ASD individuals indicated that the right cerebellum crus I activation increased with age, while the left inferior temporal gyrus activation decreased with age.

**Limitations:**

This meta-analysis is limited in its generalization of the findings to individuals with ASD by the restricted age range, heterogeneous study sample, and the large within-group variation in MNS activation patterns during object observation. Furthermore, we only included action observation studies which might limit the generalization of our results to the imitation deficits in ASD. In addition, the relatively small sample size for individual studies might also potentially overestimate the effect sizes.

**Conclusion:**

The MNS is impaired in ASD. The abnormal activation patterns were found to be modulated by the nature of stimuli and age, which might explain the contradictory results from earlier studies on the “broken mirror neuron” debate.

Autism spectrum disorder (ASD) is a pervasive neurodevelopmental disorder that affects 1 in 59 people worldwide [1]. Individuals with ASD are characterized by social communication deficits, e.g., difficulties in the production and comprehension of nonverbal gestures, as well as restricted and repetitive interests, that manifest very early in their lives. Such deficits significantly impair their social and occupational functioning [2], with previous studies showing that greater dysfunction in social communication skills predicted reduced participation in social and recreational activities in adolescence and adulthood [3]. In the past decade, researchers have attempted to identify critical components underlying lifelong social communication difficulties in ASD, one of which is the deficit in imitation.

Imitation, defined as the ability to simultaneously observe and replicate an action displayed by another person [4, 5], has been considered an important skill for early social and intellectual development [6]. Research with typically developing (TD) individuals has revealed that imitative skills progressively develop from the imitation of simple actions to the imitation of complicated gestures in the first 2 years of life as a child continuously interacts with the environment [7]. This developmental trajectory of imitation, however, has been found to be delayed in individuals with autism [8]. Moreover, imitation is a form of social learning, and deficits in imitation was found to be a significant predictor of communication development and intellectual outcomes in children with ASD [9, 10]. These studies collectively suggest that impaired imitation early in life contributes to the behavioral manifestations in ASD.

It has been proposed that the mirror neuron system (MNS) supports the ability to imitate in humans [4, 11]. The MNS is a neural circuit involving interconnected brain regions that process information related to the perception and execution of biological motions [12, 13]. Although the definition of MNS brain regions is sometimes debatable [14], some regions are well-established to be considered as the components of MNS, including the premotor cortex, inferior frontal gyrus, and inferior parietal lobule [11]. Some of these brain regions, i.e., supplementary motor area (part of the premotor cortex), have been shown to contain mirror neurons with single-cell recording [15], in which neurons in these brain regions were found to discharge both “when individuals perform a given motor act and when individuals observe another person performing a motor act with a similar goal” [16]. With the use of functional magnetic resonance imaging (fMRI) adaptation method (i.e., the ability of a brain region to adapt to repetitively displayed stimuli [17]), researchers have preliminarily shown that inferior frontal gyrus [18] and inferior parietal lobule [19] might also contain mirror neurons. The MNS is connected to other brain regions that support imitation by providing sensory/perceptual/affective inputs to frontal and parietal mirror neuron regions [14]. Previous research has shown that the organization of the MNS is task-specific. For example, imitation of actions without emotional components involves the MNS, as well as superior temporal sulcus and visual cortex for visual processing [14, 20]. Imitation of actions with emotional components, in contrast, might recruit different brain regions when compared with imitating actions without emotions [11]. For instance, cortical regions for face processing (i.e., fusiform face area [21]) and visual attention/attentional control to face stimuli (i.e., inferior occipital gyrus [22] /mid-cingulate cortex [23]), as well as subcortical regions for emotional processing (e.g., amygdala [24]) were found to be coactivated with MNS during the imitation of facial emotional expressions.

Given that the MNS is a plausible neural correlate for imitation, individuals with ASD, who have been found to have impairments in imitation, has been hypothesized to show a dysfunctional MNS. A number of fMRI studies have been conducted to compare the activation patterns of the MNS in ASD individuals with age- and/or IQ-matched TD controls during the observation of biological motions. The visual stimuli used in these fMRI studies can largely be classified into two categories: 1) biological motions without social-emotional component, such as object manipulation with hands [25] or feet [26] and 2) biological motions with social-emotional components, such as human faces or bodily gestures expressing different emotions [27–29]. These fMRI studies, however, have presented inconsistent results. For example, in a study in which images without social-emotional components were presented to participants, greater activation in the right dorsal premotor cortex (a brain region with mirror neurons) was recorded in ASD participants than in TD participants [30]. A similar action observation paradigm was adopted by Pokorny et al. [25], although they did not find significant differences in brain activation within the MNS between ASD and TD participants. With respect to the observation of stimuli with social-emotional components, there have also been inconsistencies. For example, Kim et al. [27] and Sato et al. [28] displayed both happy and fearful faces to participants with and without ASD. Kim et al. [27] reported that ASD individuals exhibited a reduction in activation in some MNS regions (i.e., inferior frontal gyrus and amygdala) in the right brain only, while Sato et al. [28] reported reductions in bilateral activation in these regions. These contradictory results have led us to two important questions regarding MNS function in individuals with ASD: Is the MNS truly dysfunctional in individuals with ASD? If their MNS is dysfunctional, how can the previous contradictory findings be explained?

Summarizing the available data with meta-analytic methods would be helpful for us to answer these questions. To our knowledge, one relevant meta-analysis has been conducted. Rather than including all MNS studies regardless of the nature of stimuli (with/without social-emotional components), this meta-analysis included only the data from action observation and imitation studies without social-emotional components among adolescents and adults (mean age = 12–33) with and without ASD using the activation likelihood estimation (ALE) method [31]. From the 13 (including four imitation and nine action observation studies) studies, the meta-analytic data revealed greater activation in the ASD than TD individuals at the right anterior inferior parietal lobule, a brain region with mirror neurons. This study appeared to provide some evidence that part of the MNS might be dysfunctional in individuals with ASD during action processing without social-emotional components. However, whether there is a deficit in MNS regardless of the nature of stimuli remains unclear. Additionally, having found that the neural network required for different visual stimuli might account for the discrepancies in fluctuating behavioral performance in individuals with ASD [32, 33], it is reasonable to postulate that the nature of stimuli presented to trigger MNS activities may play a role in explaining the inconsistent results. Furthermore, provided that the gray matter volumes in frontal, parietal, and occipital regions, where mirror neurons are situated, atypically decline in ASD compared with TD individuals starting from early adolescence (age 10–15) through adulthood [34], the age of participants across different studies may be another factor modulating the inconsistent results. An updated meta-analysis including all fMRI studies that investigated the MNS in ASD would thus be essential to draw conclusions regarding these unanswered questions, as well as to suggest hypotheses to guide future studies.

This meta-analysis aimed to explore the differences in MNS activation patterns between TD and ASD individuals when they observe/imitate biological motions with/without emotional components. Effect size signed differential mapping (ES-SDM), a mixed voxel-based meta-analytic method, was adopted [35] to synthesize the available fMRI data.

It was hypothesized that the MNS activation patterns were different between TD and ASD individuals; such differences in activation patterns would be modulated by the nature of the stimuli (i.e., biological motions with/without social-emotional components) and age (i.e., adolescent/adult). Meta-regressions, enabled by the ES-SDM, were also performed to explore clusters that exhibited statistically significant changes in activation across ages in ASD and TD individuals.

## Methods

### Literature search

This study was conducted in accordance with the Preferred Reported Items for Systematic Reviews and Meta-Analyses (PRISMA) guideline [36]. A literature search was conducted from August to October 2019 by two research assistants (I.C. and T.C.); a second search was then conducted around 1 month before this review was submitted for publication (i.e., 7 January 2020) to ensure that the data included in this study were as up-to-date as possible. To identify relevant studies, the electronic databases PsycINFO, Scopus, PubMed, Embase, Web of Science, and ScienceDirect were used with the primary keywords “mirror neuron”, “mirroring”, “action observation”, and “imitation” and secondary keywords “autism”, “autistic”, “autism spectrum disorder”, “autism spectrum condition”, “ASD”, and “ASC” together with tertiary keywords “functional magnetic resonance imaging” and “fMRI”. No limit was set to the publication dates. A manual search of the reference lists in previously published review papers [14, 16, 31, 37, 38] was also conducted to identify possible studies for the current review.

### Study inclusion

The article screening process was divided into three phases. Duplicated records were first removed, followed by the screening of title and abstracts of the remaining records to identify relevant studies. The following exclusion criteria were applied during title and abstract screening: 1) nonhuman studies, 2) treatment-related studies, 3) studies without an English version of the full text, 4) studies without empirical findings (e.g., book chapters, study protocols, and review papers), 5) studies that did not contain an experimental group with participants diagnosed with ASD and a healthy control group, 6) non-fMRI studies, and 7) resting fMRI studies. Finally, full texts of the included studies were further assessed for the eligibility of being included in the meta-analysis. Studies were excluded if 1) peak coordinates/raw statistical parametric maps could not be obtained from the published papers/contacted authors or 2) analyses were limited to specific regions of interest (ROIs). For included studies that reported results from more than one action observation experiments, we extracted and pooled the results from all eligible experiments into our analysis [39]. Additionally, although the meta-analysis of the human brain networks has provided evidence for common neural correlates of action observation and action imitation, the activation patterns related to action observation and imitation differed within the MNS regions [37]. Within this context, we believed that action observation and imitation studies should be analyzed separately. Based on our preliminary search, we observed that the number of imitation studies would not be sufficient to conduct separate analysis with adequate power, which is an important factor to be considered in conducting coordinate-based meta-analysis [40], we have excluded 6 studies [41–46] that only presented results on action imitation. The above screening processes were independently conducted by the first author and an experienced research assistant (I.C.), and their decisions were recorded on separate Excel spreadsheets. When there were discrepancies, the second author made the final decision.

### Data extraction and recoding

The demographic data, experimental details, and fMRI data of the included papers were extracted and entered into a database by the first author and checked by the second author to minimize errors. Demographic data included the sample size of each experimental and control group, mean IQ, mean age, ratio of sex (female:male), and group-matching criteria. The mean age was then recoded into two age groups, namely the adolescent group (mean age of participants 17:11 or below) and the adult group (studies recruited participants aged 18 or above). Experimental details included the descriptions about the action observation stimuli presented in the individual experiments, the body parts presented in the stimuli, and the baseline comparison. To compare the brain activation between ASD and TD individuals separately for observation of stimuli with and without social-emotional component, we classified the studies into these categories based on the following criteria: a study was classified under the subgroup “without social-emotional component” if it 1) did not present any body parts that convey emotional expressions in the visual stimuli (i.e., non-emotional condition) and 2a) did not involve more than one actor in the visual stimuli or 2b) did not present communicative (e.g., waving and hand-shaking) gestures (i.e., non-social condition) [47]. If a study failed to meet any one or both of the criteria, it was grouped under the subgroup “with social-emotional component”. Notably, if a study presented neutral facial expression as the stimuli, we classified it under the subgroup “with social-emotional component”, as suggested by previous studies which indicated that neutral faces might in fact be associated with positive or negative emotions [48, 49].

### Data analysis

All meta-analytic procedures were performed using the ES-SDM software [35].To compare the overall differences in MNS activation patterns between the ASD and TD individuals during the observation of stimuli with and without emotional components, all data from individual studies were pooled and meta-analyzed using the maximum likelihood random effects model. To evaluate the effects of the nature of tasks in the differences in MNS activation patterns between patient and control groups, random effects analyses were performed between the two groups for the subgroups of studies presented visual stimuli “with social-emotional component” and “without social-emotional component”. To evaluate the effects of age on the between-group differences in MNS activation patterns, data from studies were meta-analyzed separately for the “adolescent” and “adult” subgroups. Considering the known effects of age [34], intelligence quotient (IQ [50]), and gender [51] that contributed to the heterogeneity of the ASD population, as well as the different body parts involved in various studies that might possibly confound the brain activation [37], we conducted supplementary analyses that included age, IQ, gender, and the body part involved in the visual stimuli as covariates to control for the effects of these factors on brain activations in ASD and TD. All of the aforementioned analyses were performed by subtracting the activation map of the ASD group by the activation map of the TD group (i.e., ASD–TD contrast). To supplement the evaluation of age effects on MNS activation, brain regions that exhibited significant changes in activation within the ASD group at both adolescent and adult subgroups were explored by performing meta-regression with the mean age of participants of each study. This was a simple linear regression weighted by the squared root of sample size and restricted to predict only possible SDM values (i.e., from − 1 to 1) in the observed range of values of the chosen variable (i.e., participants’ mean age [52];). As in previous studies, the significance level of the main analyses was kept at *p* < 0.005, which requires a peak *Z* > 1 with a cluster size of 10 voxels, as suggested by Radua et al. [35] to optimize the sensitivity of results while controlling for type I error. The significance level of the meta-regression analyses was kept at *p* < 0.0005 to avoid false-positive results [53]. To evaluate the risk of reporting bias across studies, test for funnel plot asymmetry was conducted. This test examined whether the relationship between an estimated effect size and study size was greater than chance [54]. Funnel plots were generated for visual inspection of potential publication bias. In the presence of publication bias, the plot would be symmetrical at the top and data points would increasingly be missing from the middle to the bottom parts of the plot [55]. Egger’s tests [56] were then performed for the peak coordinates of brain regions showing differences between ASD and TD during action observation. Significant Egger’s tests indicate “small study effects”. Sometimes, smaller studies would in fact yield larger effects than studies with a larger sample size [57], this might be due to publication bias [58].

## Results

### Study selection

**Fig. 1:**
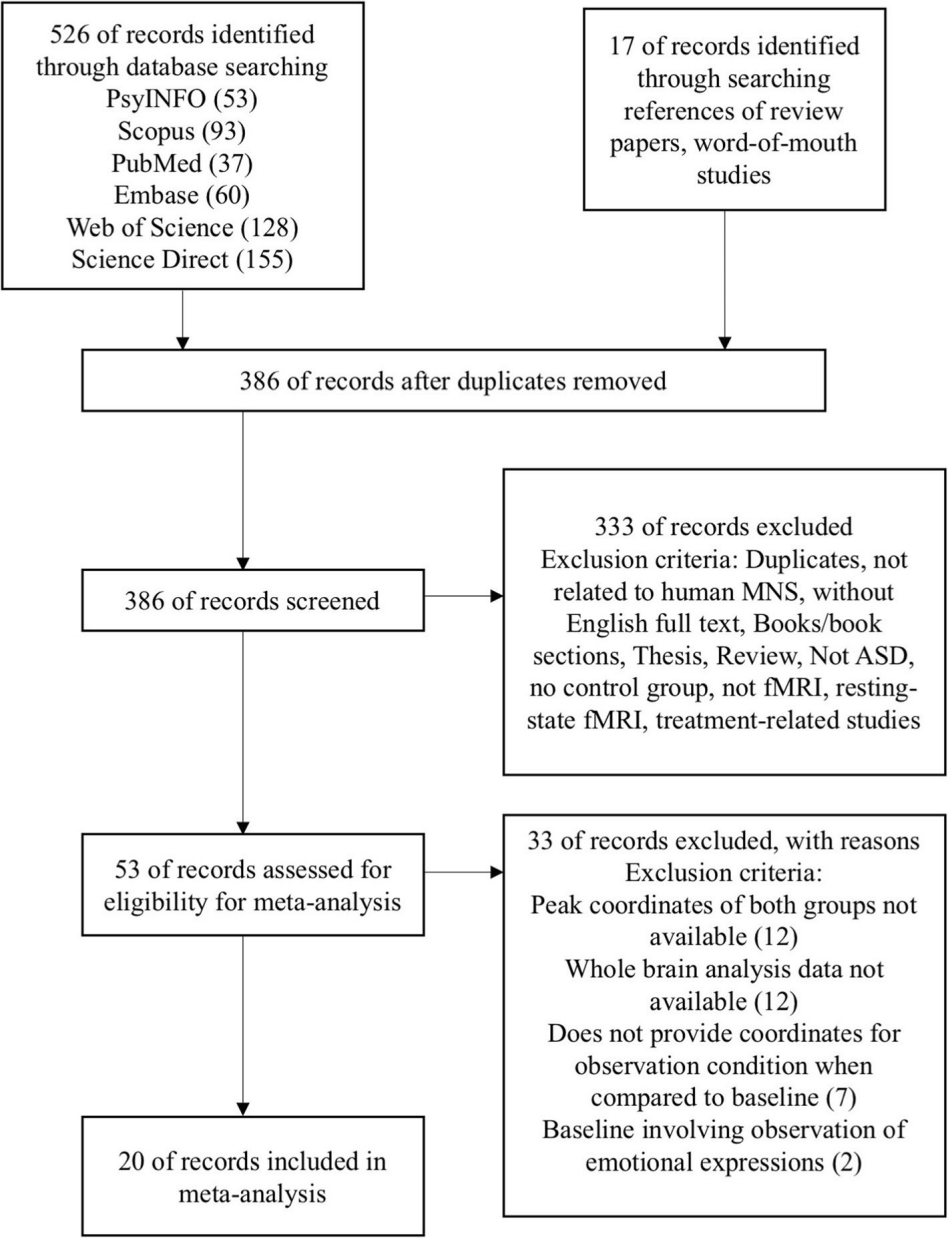
Flowchart of the article screening process

A total of 543 studies were retrieved. After 157 duplicate records were removed, titles and abstracts of 386 records were screened. With exclusion criteria applied, 326 records were excluded, with 60 records remaining for the full-text screening. During full-text screening, 40 records were further excluded. The complete process of article selection is outlined in Fig. 1.

### Characteristics of the included studies

A total of 20 action observation studies (including 24 experiments) were included in the meta-analysis, which represented 284 individuals with ASD (111 adolescents and 173 adults) compared with 290 matched, typically developing controls (114 adolescents and 176 adults). Nine studies (with nine experiments) presented biological motions without social-emotional components [59–67], and the remaining 13 studies (with 15 experiments) presented biological motions with social-emotional components [62, 65, 68–78]. The demographic data of the participants and the experimental details of each study are summarized in Table 1.

**Table 1:** Twenty fMRI action observation studies (with 24 experiments) included in the meta-analysis

Demographic Data									Experimental Design			
Study	Sub-groups	Sample size	Age group	Mean IQ (SD)	Ratio of sex (F:M)	Symptom severity measure	Symptom severity score (SD)	Subject matching criteria	Action observation stimuli	Body part	Social-emotional component	Baseline
Bastiaansen(2011) [68]	ASD-HTD	2121	Adult	102.5 (14.81)101.5 (17.4)	0:210:21	ADOS (social)	7.19 (2.46)	Age, IQ, gender	One actor displayed facial expressions (happy, neutral, disgusted)	Face	Yes	Fixation cross
Bookheimer (2008) [69]	ASD-HTD	1212	Adolescent	N/A	0:120:12	ADOS (total)	11.3 (N/A)	Age, gender	One actor displayed a facial expression (neutral)	Face	Yes	Matching oval shapes
Carter (2012) [78]	ASD-HTD	1213	Adolescent	112.1 (15.19)116.6 (10.28)	3:92:11	ADOS (social)	7.25 (1.22)	Age, IQ, gender	Two actors displayed bodily gestures in social situations	Full-body	Yes	Fixation cross
Cole (2019) [59]	ASD-HTD	2020	Adult	N/A	8:128:12	AQ	37.4 (8.04)	Age, gender, education	One actor manipulated an object (inserting poker chips into a box)	Full-body	No	Fixation cross
Critchley (2000) [70]	ASD-HTD	99	Adult	102 (15)116 (10)	0:90:9	ADI	N/A	Age, IQ, gender	One actor displayed facial expressions (happy, neutral, angry)	Face	Yes	Observation of neutral face
Dalton (2005) [71]	ASD-HTD	1212	Adolescent	94 (19.47)N/A	0:120:12	ADI-R	N/A	Age, gender	One actor displayed facial expressions (happy, fear, angry)	Face	Yes	Observation of neutral face
Dapretto (2006) [72]	ASD-HTD	55	Adolescent	92.0 (19.4)105.2 (12.8)	0:50:5	ADOS (social)	8.4 (3.1)	Age, IQ, gender	One actor displayed facial expressions (happy, sad, angry, fear, neutral)	Face	Yes	Fixation cross
Davies (2011) [73]	ASD-HTD	1616	Adolescent	106.2 (20.3)105.6 (16.0)	2:142:14	ADOS (total)	12 (4)	Age, IQ, gender	One actor displayed facial expressions (happy, angry, fear, neutral)	Face	Yes	Fixation cross
Deeley (2007) [74]	ASD-HTD	99	Adult	114 (12)120 (18)	0:90:9	ADOS; ADI-R	N/A	IQ, gender	Experiment 1:One actor displayed a facial expression (fear)	Face	Yes	Fixation cross
									Experiment 2:One actor displayed a facial expression (disgust)			
									Experiment 3:One actor displayed a facial expression (happy)			
Freitag (2008) [60]	ASD-HTD	1313	Adolescent	101.2 (21.2)112.1 (18.0)	2:132:13	ADI-R (social)	11.3 (4.3)	Age, IQ, gender	One point-light walker displayed bodily gesture (walking)	Full-body	No	Scrambled point-light displays
Greimel (2010) [75]	ASD-HTD	1515	Adolescent	112.7 (11.3)109.9 (17.3)	0:150:15	ADOS, ADI-R	N/A	Age, IQ, gender	One actor displayed facial expressions (happy, sad)	Face	Yes	Neutral faces
Grèzes (2009) [61]	ASD-HTD	1212	Adult	102 (20.6)119 (6.6)	2:100:12	N/A	N/A	Age, IQ, gender	One actor displayed bodily emotional expression (fear)	Full-body	Yes	Neutral bodily expression
Hubbard (2012) [62]	ASD-HTD	1313	Adolescent	110116	3:103:10	ADOS (social)	7.9 (3)	Age, IQ, gender	Experiment 1:One actor displayed a random hand gesture	Hand	No	Still frame (a kitchen) without presence of hands
									Experiment 2:One actor (without face) displayed communicative gesture	Hand	Yes	
Libero (2014) [63]	ASD-HTD	2122	Adult	116.3 (12.8)117.5 (8.8)	4:175:17	ADOS, ADI-R	N/A	Age, IQ, gender	One actor manipulated objects (telephone, spoon, camera, cup)	Full-body	No	Fixation cross
Marsh (2011) [64]	ASD-HTD	1819	Adult	110.2 (16.0)113.9 (13.8)	N/A	ADOS (social)	6.17 (3.47)	Age, gender	One actor manipulated an object (a ball)	Hand	No	Moving shapes without hand action
Martineau (2010) [66]	ASD-HTD	78	Adult	93.3 (9.5)N/A	0:70:8	N/A	N/A	Age, education	One actor displayed hand flexion-extension movement	Hand	No	Picture of a static hand
McKay (2012) [67]	ASD-HTD	1010	Adult	125 (7.01)124.8 (6.75)	N/A	N/A	N/A	Age, IQ	One point-light walker displayed bodily gesture (walking)	Full-body	No	Scrambled point-light displays
Schneider (2013) [76]	ASD-HTD	2828	Adult	109.1 (9.2)114.0 (9.6)	13:1513:15	AQ	37.69 (7.84)	Age, gender, education	One actor displayed facial expressions (disgust, happy, fear, anger)	Face	Yes	Neutral facial expression
Schulte-Ruther (2011) [77]	ASD-HTD	1818	Adult	106.6 (10.5)112.1 (10.4)	0:180:18	AQ	33.6 (10.2)	Age, IQ, gender	One actor displayed facial expressions (happy, sad)	Face	Yes	Neutral facial expression
Wadsworth (2018) [65]	ASD-HTD	1315	Adolescent	109 (17.3)102 (16.2)	2:114:11	AQ	72 (34.36)	Age, IQ	Experiment 1:One actor manipulated daily objects (e.g., an iron)	Full-body	No	Fixation cross
									Experiment 2:More than one actors displayed communicative gestures	Full-body	Yes	

*ASD-H* High-functioning autism spectrum disorder, *TD* Typically-developing, *ADOS* Autism Diagnostic Observation Schedule, *ADI-R* Autism Diagnostic Interview—Revised, *Age* chronological age only, *IQ* full-scale IQ only, *Education* years of education

### Global differences in MNS activation patterns between ASD and TD individuals during action observation (Table 2)

**Fig. 2:**
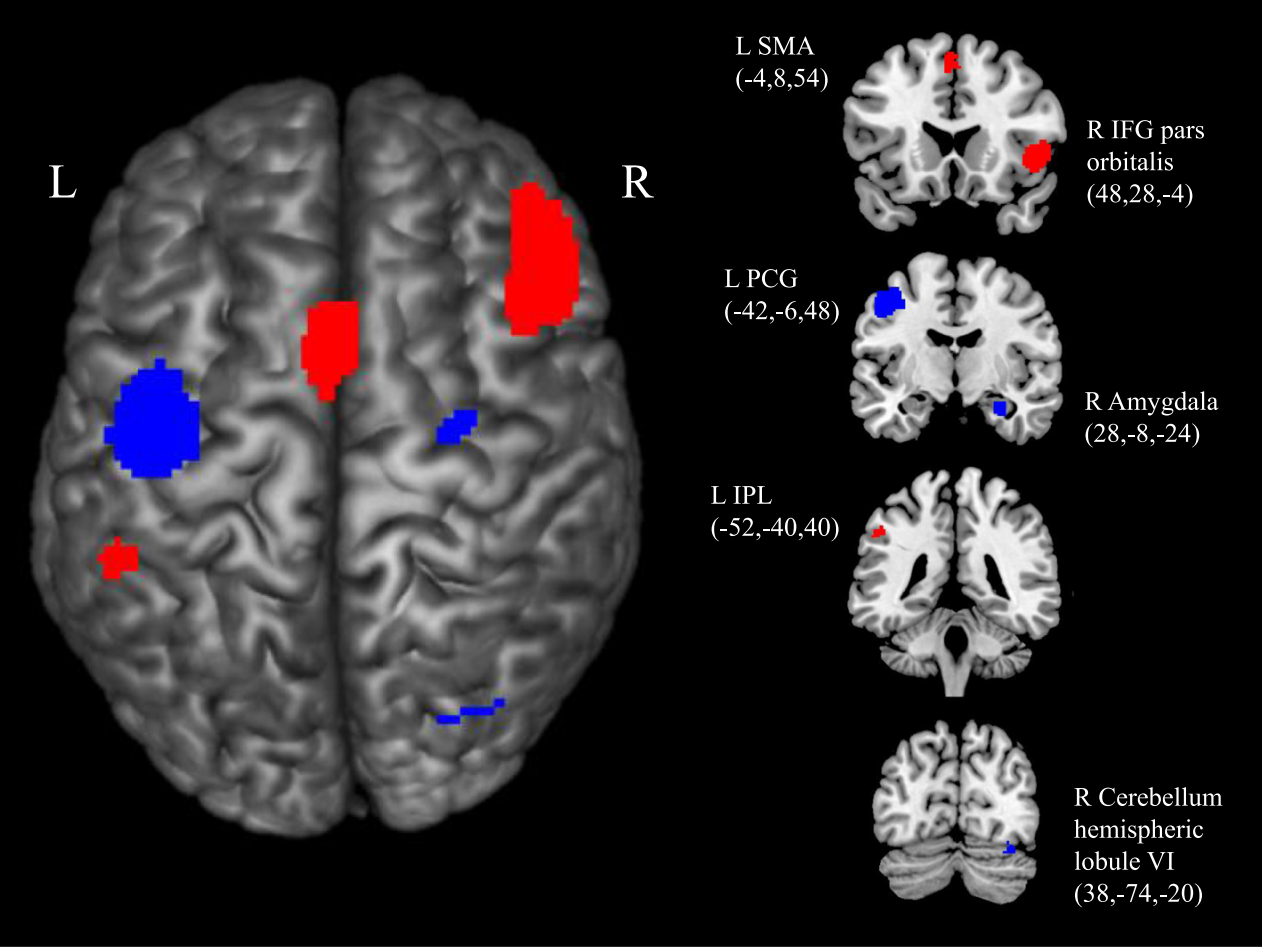
Difference in MNS activation between ASD and TD during action observation. Clusters highlighted in red indicates hyperactivation in ASD when compared with TD; clusters highlighted in blue indicates hypoactivation in ASD when compared with TD (*p* < .005, uncorrected). *(*Note: *L =* left*, R =* right, *SMA =* supplementary motor area, *IFG =* inferior frontal gyrus, *PCG*=precentral gyrus, *IPL*=inferior parietal lobule)

During the observation of actions with and without social-emotional components, it was found that people with autism exhibited hyperactivation in the right inferior frontal gyrus (orbital part; BA45, BA47), left supplementary motor area (BA6) and left inferior parietal lobule (BA40; brain clusters highlighted in red in Fig. 2). In contrast, hypoactivation was observed in the cluster of the left precentral gyrus (BA6), right amygdala and right cerebellum hemispheric lobule VI (brain clusters highlighted in blue in Fig. 2). All clusters remained statistically significant (ps < 0.005) with ratio of sex, mean age, or body parts as covariates. Moreover, with the exception of left inferior parietal lobule, our results remained significant when mean IQ or both mean age and mean IQ were included as covariates.

**Table 2:** Global differences in MNS activation during action observation

Brain regions with peak activation						Cluster breakdown
Anatomical region	L/R	Total number of voxels	MNI coordinates	SDM-Z	*p* (uncorrected)	Anatomical regions (Broadmann area)
*ASD > TD*						
Inferior frontal gyrus, orbital part	R	1077	48,28, − 4	2.827	~0	Inferior frontal gyrus, orbital part (BA47) Inferior frontal gyrus, orbital part (BA45) Inferior frontal gyrus, triangular part (BA45)
Supplementary motor area	L	525	− 4,8,54	2.044	< 0.0005	Supplementary motor area (BA6)
Inferior parietal lobule	L	46	− 52, − 40,40	1.710	< 0.005	Inferior parietal lobule (BA40)
*ASD < TD*						
Precentral gyrus	L	692	− 42, − 6,48	− 2.169	< 0.0001	Precentral gyrus (BA6) Postcentral gyrus (BA6)
Amygdala	R	68	28, − 8, − 24	− 1.451	< 0.005	Amygdala (BA34) Hippocampus (BA20) Median cingulate
Cerebellum, hemispheric lobule VI	R	17	38, − 74, − 20	− 1.392	< 0.005	Cerebellum, hemispheric lobule VI (BA19) Cerebellum, crus I (BA19)

### Effects of nature of stimuli on the differences in MNS activation patterns in ASD and TD individuals (Tables 3 and 4)

**Fig. 3:**
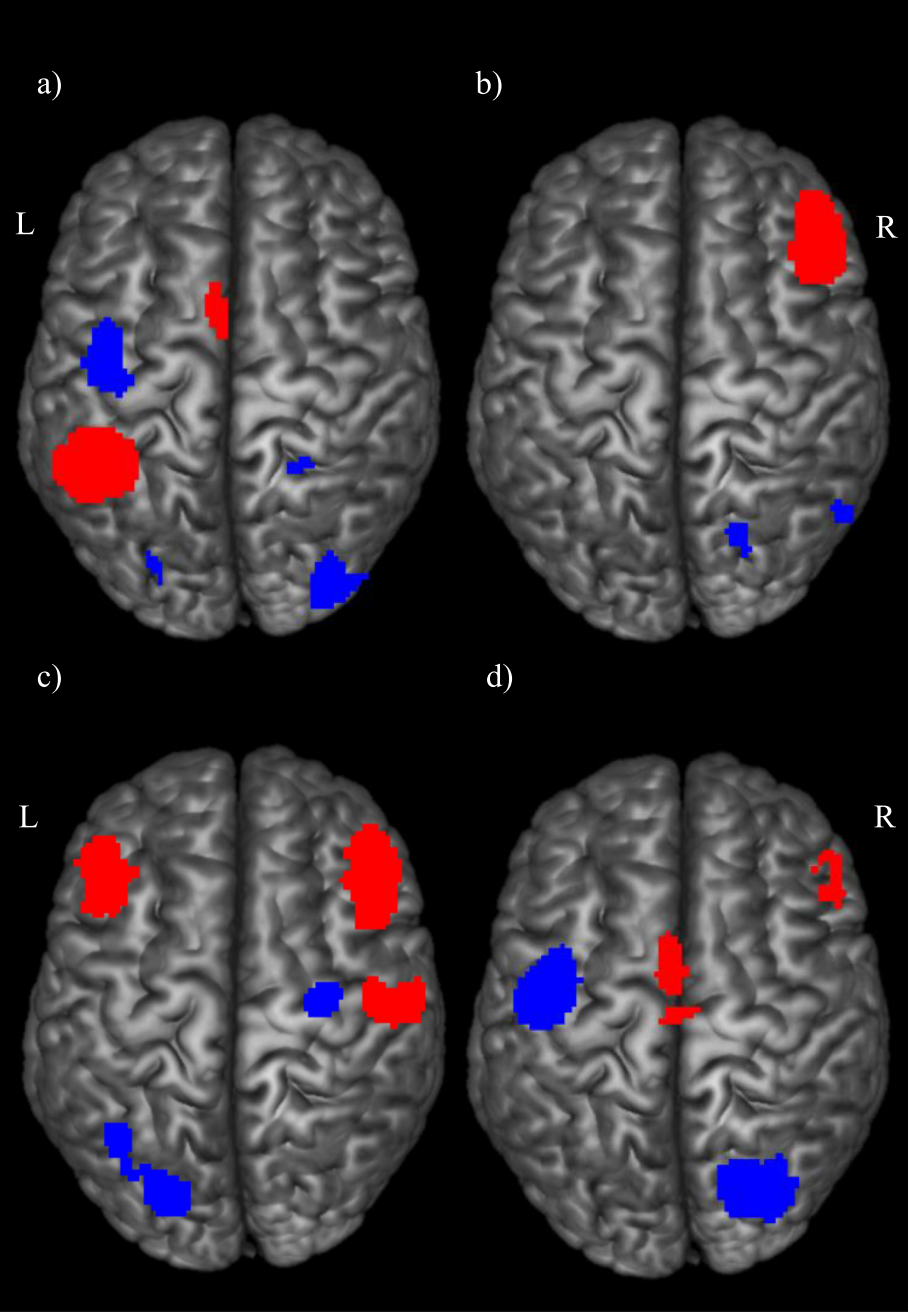
Effects of nature of stimuli and age in MNS activation between ASD and TD. Each figure corresponds to the following condition: **a** action observation without social-emotional components, **b** action observation with social-emotional components, **c** action observation in the adolescent subgroup, **d** action observation in the adult subgroup. Clusters highlighted in red indicates hyperactivation in ASD when compared with TD; clusters highlighted in blue indicates hypoactivation in ASD when compared with TD (*p* < .005, uncorrected). (Note: *L* = left, *R* = right)

During the observation of stimuli without social-emotional components, the left inferior parietal lobule (significant cluster that extended to the supramarginal gyrus) and left supplementary motor area were hyperactivated in the ASD compared with TD individuals. Meanwhile, the individuals with ASD also showed hypoactivation in the middle occipital gyrus, bilateral postcentral gyrus, and left cerebellum crus I (Fig. 3a). All clusters remained statistically significant (ps < 0.005) with ratio of sex, mean age, mean IQ, or body parts as covariates. Additionally, with the exception of right postcentral gyrus and left cerebellum crus I, our findings remained significant when mean age and mean IQ were included as covariates.

**Table 3:** Difference in MNS activation between ASD and TD during observation of stimuli without social-emotional components

Brain regions with peak activation						Cluster breakdown
Anatomical region	L/R	Total number of voxels	MNI coordinates	SDM-Z	*p* (uncorrected)	Anatomical regions (Broadmann area)
*ASD>TD*						
Inferior parietal lobule	L	1036	− 42, − 50, 48	2.382	< 0.0001	Inferior parietal lobule (BA40)
Supplementary motor area	L	185	− 4, 4, 54	1.997	< 0.001	Supplementary motor area (BA6)
*ASD<TD*						
Middle occipital gyrus	R	368	32, − 84, 2	− 2.172	< 0.0005	Middle occipital gyrus (BA18) Middle occipital gyrus (BA19) Inferior network, inferior longitudinal fasciculus
Postcentral gyrus	L	306	− 42, − 14, 48	− 1.950	< 0.0005	Precentral gyrus (BA6) Postcentral gyrus (BA6)
Postcentral gyrus	R	30	22, − 46, 60	− 1.451	< 0.005	Postcentral gyrus (BA2)
Cerebellum, crus I	L	20	− 26, − 82, − 24	− 1.428	< 0.005	Cerebellum, crus I (BA19)

**Table 4:** Difference in MNS activation between ASD and TD during observation of stimuli with social-emotional components

Brain regions with peak activation						Cluster breakdown
Anatomical region	L/R	Total number of voxels	MNI coordinates	SDM-Z	*p* (uncorrected)	Anatomical regions (Broadmann area)
*ASD>TD*						
Inferior frontal gyrus, orbital part	R	1229	50, 28, − 4	3.276	~0	Inferior frontal gyrus, orbital part (BA47) Inferior frontal gyrus, triangular part (BA45)
*ASD<TD*						
Cerebellum, hemispheric lobule VI	R	15	20, − 70, − 22	− 1.567	< 0.005	Cerebellum, Hemispheric lobule VI (BA18) Cerebellum, Hemispheric lobule VI (BA19)

During the observation of stimuli containing social-emotional components, highly significant hyperactivation of the MNS in the ASD individuals was found in the right inferior frontal gyrus (orbital part). In contrast, hypoactivation of the left cerebellum (hemispheric lobule VI) was observed in ASD individuals compared with the TD individuals (Fig. 3b). The hyperactivation of the right inferior frontal gyrus (orbital part) remained highly significant with ratio of sex, mean age, mean IQ, or body parts as covariates, as well as with mean age and mean IQ as covariates (all ps < 0.005). Furthermore, the hypoactivation of cerebellum remained statistically significant when IQ was included as a covariate.

### Effects of age on the differences in MNS activation patterns between ASD and TD individuals (Tables 5 and 6)

In the adolescent subgroup (ASD mean age = 11.3–17.6; TD mean age = 11.5–17.1; Fig. 3c), the ASD individuals showed hyperactivation in the bilateral inferior frontal gyrus pars orbitalis and pars triangularis, right postcentral gyrus extending to the precentral gyrus, and the left supramarginal gyrus extending to the inferior parietal gyrus (BA40, BA2). Hypoactivation in the ASD compared to TD individuals was found in the left fusiform gyrus (BA18, BA19), left cerebellum (hemispheric lobule VI), and right median cingulate around the hippocampal region (BA20). With mean age or body part as covariates, all brain clusters remained statistically significant. With ratio of sex as a covariate, all clusters except left supramarginal gyrus remained significant. With mean IQ as a covariate, all clusters except left supramarginal gyrus and left cerebellum hemispheric lobule VI remained significant. With both mean age and mean IQ as covariates, all clusters except left cerebellum hemispheric lobule VI remained significant.

**Table 5:** Difference in MNS activation between ASD and TD in adolescent subgroup

Brain regions with peak activation						Cluster breakdown
Anatomical region	L/R	Total number of voxels	MNI coordinates	SDM-Z	*p* (uncorrected)	Anatomical regions (Broadmann area)
*ASD>TD*						
Inferior frontal gyrus (orbital part)	R	927	48,30, − 8	2.441	< 0.0001	Inferior frontal gyrus, orbital part (BA47) Inferior frontal gyrus, triangular part (BA45)
Inferior frontal gyrus (orbital part)	L	484	− 44, 24, − 8	1.882	< 0.001	Inferior frontal gyrus, orbital part (BA47) Inferior frontal gyrus, triangular part (BA47)
Postcentral gyrus	R	410	62, − 14, 34	1.659	~0.001	Postcentral gyrus (BA3, BA43) Precentral gyrus (BA4,BA6)
Supramarginal gyrus	L	20	− 56, − 40, 36	1.531	< 0.005	Supramarginal gyrus (BA40, BA2) Inferior parietal gyrus (BA40)
*ASD<TD*						
Fusiform gyrus	L	300	− 24, − 82, − 18	− 1.780	< 0.001	Fusiform gyrus (BA18, BA19) Lingual gyrus (BA18)
Median cingulate	R	121	32, − 12, − 26	− 1.409	~0.001	Hippocampus (BA20) Median cingulate
Cerebellum, hemispheric lobule VI	L	56	− 38, − 62, − 26	− 1.350	< 0.005	Cerebellum, hemispheric lobule VI (BA37) Cerebellum, crus I (BA37)

**Table 6:** Difference in MNS activation between ASD and TD in adult subgroup

Brain regions with peak activation						Cluster breakdown
Anatomical region	L/R	Total number of voxels	MNI coordinates	SDM-Z	*p* (uncorrected)	Anatomical regions (Broadmann area)
*ASD>TD*						
Supplementary motor area	L	249	− 4,2,50	2.017	< 0.0005	Supplementary motor area (BA6, BA24)
Inferior frontal gyrus (orbital part)	R	71	54, 26, − 4	1.826	~0.001	Inferior frontal gyrus, orbital part (BA45) Inferior frontal gyrus, triangular part (BA45)
*ASD<TD*						
Precentral gyrus	L	708	− 42, 6, 48	− 2.565	~0.00001	Precentral gyrus (BA6) Postcentral gyrus (BA4, BA6)
Cerebellum, hemispheric lobule VI	R	742	18, − 72, − 28	− 1.987	< 0.001	Cerebellum, hemispheric lobule VI (BA18) Cerebellum, crus I (BA19)

In the adult subgroup (ASD mean age = 23–37; TD mean age = 21–37; Fig. 3d), the right inferior frontal gyrus (orbital part extending to the triangular part) and left supplementary motor area (BA6, BA24) were hyperactivated, whereas the left precentral gyrus extending to the postcentral gyrus (BA6, BA4), and the right cerebellum (hemispheric lobule VI extending to crus I) were hypoactivated in the ASD compared with TD individuals. All clusters remained statistically significant (ps < 0.005) with ratio of sex, mean age, and body parts as covariates. In addition, except for the left supplementary motor area, the findings remained significant when mean age, mean IQ, or age and IQ were included as covariates.

### Meta-regression

**Fig. 4:**
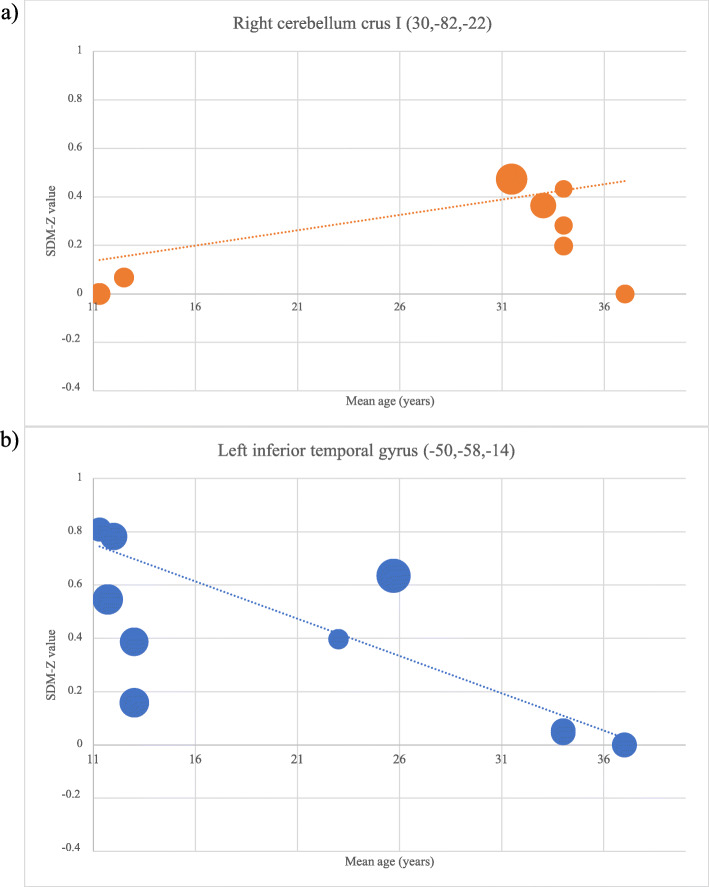
Meta-regression shows that the activation of two brain regions in ASD individuals were significantly associated with chronological age; as age increases: **a** the right cerebellar crus I was more activated and **b** the left inferior temporal gyrus was less activated. Of note, the significant correlation between increasing age and increasing activation in the right cerebellum crus I was driven by two studies (data represented by the two orange dots lying on the left end of the *x*-axis). The interpretation of this significant correlation should be treated with caution. (Note: *Studies that reported a peak in these two regions were included in the calculation; each of these studies is represented as a dot; larger dots represent larger sample sizes*)

Among the brain regions with significant differences in hyper-/hypoactivation in ASD shown in the adolescent and adult subgroups, statistically significant correlations (ps < 0.0005) were found between age and activation in the right cerebellum crus I and the left inferior temporal gyrus within ASD individuals (Fig. 4). Specifically, meta-regression of eight studies reflected that the activation of the right cerebellum crus I increased with increasing age. In contrast, meta-regression of nine studies showed that the activation of the left inferior temporal gyrus decreased with increasing age.

### Risk of publication bias

**Fig. 5:**
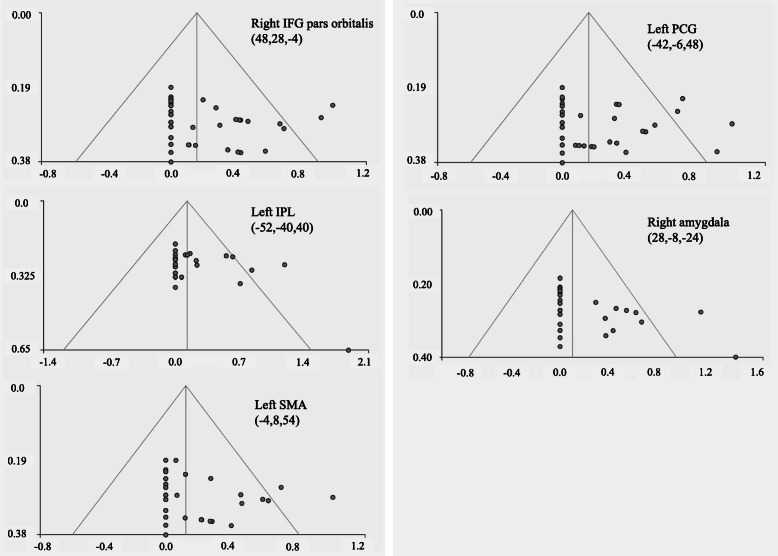
Funnel plots of the activation effect sizes in various brain regions showing differences between ASD and TD during action observation. The horizontal axis represents the effect size patients and controls. The vertical axis represents the standard error. Publication bias might be present in the left inferior parietal lobule and right amygdala clusters. (Note: *IFG* = inferior frontal gyrus; *IPL* = inferior parietal lobule; *SMA*= supplementary motor area; *PCG* = precentral gyrus)

For each individual study, the estimated effect sizes of the peak coordinates showing statistically significant differences between the ASD and TD groups (listed in Table 2) were extracted to generate the funnel plots (Fig. 5). Visual inspection of the funnel plots of some MNS regions (Fig. 5a, c, d) did not show obvious publication bias. Conversely, publication bias might be present in the left inferior parietal lobule and right amygdala (Fig. 5b, e), evidenced by the presence of relatively symmetrical appearance at the top, with more study data missing from the middle to the bottom of these graphs. Egger’s tests remained non-significant for the right inferior frontal gyrus *pars orbitalis* (*p* = 0.428), left supplementary motor area (*p* = 0.182), and left precentral gyrus (*p* = 0.551), approached significance for the left inferior parietal lobule (*p* = 0.080), and reached statistical significance for the right amygdala (*p* = 0.024). Collectively speaking, these results indicated that small study effects, which might be due to publication bias, were observed in the left amygdala cluster in the main analysis.

## Discussion

This meta-analysis sought to identify the activation pattern of MNS during observation of stimuli with/without social-emotional components among individuals with ASD compared with age-matched typically developing controls. After a comprehensive literature search and the application of inclusion and exclusion criteria, 20 action observation studies (with 24 experiments) were included in the meta-analysis. In summary, this coordinate-based fMRI meta-analysis indicated three main points. First, the MNS is impaired in ASD during action observation; second, MNS dysfunction in ASD individuals is modulated by social-emotional components of the visual stimuli during action observation. Third, age seems to be an important factor in MNS function in ASD. The following discussion is divided into three parts, with each part discussing the possible implications of the points listed above.

### Abnormal activation within and beyond MNS in ASD

There has been a hot debate in the past decade on whether mirror neurons are “broken” in ASD, leading to the impairment in imitation in this group of individuals. The “broken mirror neuron theory” for autism asserts that the frontal and parietal brain regions with mirror neurons were found to be abnormally activated in individuals with ASD and given the property of mirror neurons (i.e., discharges during both activation observation and execution) that appears to support imitation, impaired imitation might be associated with the “broken” frontoparietal mirror neurons [38, 72]. This meta-analysis indeed demonstrated abnormal MNS activations in individuals with ASD, evident by the hyperactivation of the right inferior frontal gyrus and left supplementary motor area, which supports the “broken mirror neuron theory”. However, at the same time, abnormal activations were also shown in brain regions beyond MNS, that is the hypoactivation of left precentral gyrus, amygdala, and cerebellum. With the abnormalities shown in both MNS and other brain regions, one of the possible explanations is that the imitation deficits in ASD might not be solely attributable to the “broken mirror neurons”, but the interaction between MNS and other brain regions. One of the frameworks that studies the interplay between brain regions is neural connectivity [79]. With regards to ASD, the disrupted connectivity theory posits that abnormal structural/functional connectivity in the brain might underlie the manifestation of behavioral phenotypes in ASD [80]. Numerous empirical studies, including those using electrophysiological [81, 82] and neuroimaging [83] techniques, have pointed to disrupted neural connectivity in individuals with ASD. In particular, abnormal connectivity was found within the MNS. Regarding the neural connectivity between brain regions with mirror neurons, Rudie et al. [84] recorded hypoconnectivity between the left inferior frontal gyrus pars opercularis and left parietal cortex in individuals with ASD relative to healthy controls. Regarding the connectivity between mirror neuron regions and the supporting MNS regions, it was revealed that the amygdala was hypoconnected with the frontal regions where the MNS is situated [85]. This evidence collectively implies that atypical connectivity within the MNS contributes to dysfunction, which might be associated with the abnormal MNS activation patterns found in this meta-analysis.

### MNS impairment during the observation of stimuli with/without social-emotional components

Two findings in the comparison between ASD and TD individuals, regarding the differences in activation patterns when they observe stimuli with and without social-emotional components, confirm our hypothesis that nature of the stimuli (with/without social-emotional components) might be one of the modulators of neural activation of the MNS. First, regarding the observation of stimuli without emotions, the MNS in the left hemisphere (i.e., left inferior parietal lobule and left supplementary motor area) were found hyperactivated, while the right middle occipital gyrus and the left postcentral gyrus were found hypoactivated in ASD when compared with TD individuals. Notably, the left-lateralized hyperactivation of MNS shown in our study might be an important finding that enhances our understanding of the MNS deficits in ASD. Given an increase in neural activation measured by fMRI has been found to indicate an increase in mental effort during tasks to maintain behavioral performance [86], and the left-lateralized abnormalities in structural connectivity found in ASD in a previous study [87], it might be possible that individuals with ASD require extra mental effort for observation of stimuli without emotions, which could be attributable to the possible MNS deficits specifically in the left hemisphere. In terms of the MNS regions showing significant differences between ASD and TD, our results were largely consistent with that reported by Yang and Hofmann [31]. However, we also noted some diverging results when compared with their study. Specifically, the hypoactivation in the left cerebellum crus I and right postcentral gyrus were not found in their study, while our results reported highly significant hyperactivation in the left, but not the right, inferior parietal lobule. Given action observation and imitation was shown to engage the MNS as other brain regions in a differential manner [37], the diverging results could be due to the exclusion of the imitation studies in our meta-analysis. Furthermore, different methods of analysis and threshold levels might as well contribute to the inconsistency of results [88].

Second, regarding the observation of stimuli with emotions, our meta-analytic results indicated that the hyperactivation of the right inferior frontal gyrus in ASD individuals was statistically significant when compared with the TD individuals. Importantly, the hyperactivation of this brain region remained highly significant when mean age, mean IQ, gender, and body parts are included as covariates. Given that the right inferior frontal gyrus was considered one of the core MNS regions [11, 14], our results might imply that the impaired right frontal MNS could possibly be associated with the dysfunctional social-emotional processing, one of the key characteristics of ASD [89]. Indeed, among healthy individuals, it has been shown that the right inferior frontal gyrus is one of the possible neural correlates of social-emotional perception [90–93], in which its connectivity with the limbic system predicts the individual differences in successful emotional regulation [94]. The brain–behavior relationship between the right inferior frontal gyrus and social-emotional processing in ASD could be further investigated to confirm our speculation.

### MNS activation in different age groups

Another set of analyses we performed was to investigate the activation patterns of MNS and other brain regions in the adolescent and adult subgroups. In addition, we conducted meta-regression with mean age as the regressor within the ASD group to preliminarily observe the MNS developmental trajectory in this group of individuals. In the adolescent subgroup, bilateral frontoparietal MNS were shown to be hyperactivated in the individuals with ASD; the extent of this hyperactivation was shown to remain only in the frontal MNS regions (i.e., left supplementary motor area and right inferior frontal gyrus pars orbitalis) in the adult subgroup. One explanation for the difference in the activation patterns of MNS between the adolescent and adult subgroups could be due to the greater variation in the adult subgroup, which in turn may reduce the statistical significance of the observed effects. Notwithstanding this limitation, our results provided preliminary evidence in favor of the age effect on MNS functioning in ASD. Further research is necessary to substantiate the present findings and examine the role that age plays on the MNS functioning in ASD.

In addition, using the linear meta-regression method, we identified a significant linear decrease in the activation of the left inferior temporal gyrus as age increased in the ASD group. The meta-analytic results also reflected that hyperactivation in the left temporal gyrus was only shown in the adolescent subgroup but not in the adult subgroup. This echoed the results in a previous study, in which the inferior temporal gyrus gray matter volume changed from leftward asymmetry (i.e., thicker gray matter on the left side) toward symmetry in individuals with autism from childhood through adulthood [95]. Conversely, the right cerebellum appeared to exhibit a linear age-related increase in activation. In the adolescent subgroup, the right cerebellum exhibited hyperactivation, and the degree of hyperactivation was further increased in the adult subgroup. The increase in the intensity of hyperactivation may be a consequence of increased lateralization with age [96]. Although the two brain areas are not considered to be core MNS regions, our findings seem to suggest that the age-related changes in other brain regions beyond MNS could also influence the activation of MNS. More studies are needed to delineate the reciprocal influences and interplay between the inferior temporal gyrus, cerebellum, and the MNS over time.

### Limitations

Regarding literature search, we have attempted to obtain a comprehensive set of relevant fMRI data for meta-analysis by extending our scope of search. Using multiple search engines and manual searches from the reference lists of multiple previous review papers, we found 20 suitable action observation studies that provided us with whole brain analytic data for meta-analysis. Although we found 12 additional studies that could potentially be included in the meta-analysis, these studies either included only the region of interest analyses, or the whole brain analysis data were not available. Hereby, the power of this meta-analysis would have been increased, and a more comprehensive picture regarding MNS functioning in ASD could have been presented if the whole-brain results were available in the additional neuroimaging studies. Additionally, since our meta-analysis only included action observation but not imitation studies, the generalizability of the results to the imitation deficits in ASD might be limited. Nevertheless, findings from previous studies have suggested that action observation is in fact one of the key neural processes supporting imitation [11, 37]. In support of it, evidence yielded from single-neuron recording in humans has reported a three-way differentiation with MNS. For instance, the MNS contained different subtypes of mirror neurons, including neurons typically discharging both during action observation and execution, neurons that discharge only during action observation, and neurons that discharge only during action execution [15]. These results are in line with previous meta-analysis showing differential activation patterns related to action observation and imitation within the MNS regions [37]. Future fMRI meta-analysis that include more imitation studies in ASD with more homogeneous study design would help extend current knowledge of brain networks underlying impaired imitation in ASD.

In addition, the age of the participants in the included studies ranged from 11.3–37 for ASD individuals and 11.5–37 for TD individuals. Thus, the age effect on MNS can be examined within this age range only; the difference between the ASD and TD individuals in MNS activation remains unknown for populations beyond this age range. Future fMRI studies related to the MNS that target these age ranges are recommended so that the age effect on the MNS can be examined more holistically. Moreover, the meta-regression we performed using the ES-SDM software was a linear regression, which assumed the variable of interest (i.e., mean age) has a linear relationship with brain activation. However, a previous study has shown that the developmental trajectory of the ASD brain appeared to be u-shaped [34], suggesting that linear meta-regression method may not be ideal for articulating the association between age and MNS activation; together with the fact that the statistical significances found in the reported brain regions were driven by a limited number of studies, results have to be treated with caution.

Heterogeneity of IQ and gender in our study samples is another limitation as some of the MNS abnormal activations disappeared when IQ and age were included as covariates. Future studies with a more homogeneous sample should be warranted to confirm the observation in our meta-analysis. In addition, the heterogeneity of ASD symptomatology and the overrepresentation of adult age group may limit the generalization of the findings to the entire patient population with ASD. Future studies with a broader age range, and including different levels of functioning, would help to delineate the intricate relationship between IQ, age, and levels of symptom severity in wider patient population with the disorder. Furthermore, it should be noted that our included studies generally had a small sample with a range of 5 to 21 participants per group. Given that studies with small sample sizes tend to overestimate the study effect [57], it might be possible that the MNS abnormality in ASD might have been overestimated. Thus, the generalization of the findings to individuals with ASD in general may be limited by the relatively small sample size in our meta-analysis.

## Conclusion

This meta-analysis aimed to explore the differences in MNS activation patterns between TD and ASD individuals when they observe biological motions with/without social-emotional components. ES-SDM meta-analytic method was adopted to synthesize the available fMRI data. After a comprehensive literature search, whole-brain analysis data from 20 journal articles with 24 experiments were included in the meta-analysis. In summary, this study indicated that the MNS was hyperactivated in ASD, which might underlie the imitation impairments in these individuals. The abnormal activation patterns were modulated by the nature of stimuli and age, which might explain the previous peculiar results contributing to the unresolved “broken mirror neuron” debate.

## Supplementary information

**Additional file 1: Table S1.** PRISMA checklist.

## Data Availability

Not applicable

## Abbreviations

ALE: Activation likelihood estimation
ASC: Autism spectrum condition
ASD: Autism spectrum disorder
BA: Brodmann area
ES-SDM: Effect size signed differential mapping
fMRI: Functional magnetic resonance imaging
MNS: Mirror neuron system
ROIs: Regions of interest
TD: Typically developing

## References

[1] Baio J, Wiggins L, Christensen DL, Maenner MJ, Daniels J, Warren Z (2018). Prevalence of autism spectrum disorder among children aged 8 years—autism and developmental disabilities monitoring network, 11 sites, United States, 2014. MMWR Surveill Summ..

[2] American Psychiatric Association (2013). Diagnostic and Statistical Manual of Mental Disorders.

[3] Orsmond GI, Krauss MW, Seltzer MM (2004). Peer relationships and social and recreational activities among adolescents and adults with autism. J Autism Dev Disord..

[4] Heyes C (2011). Automatic imitation. Psychol Bull..

[5] Chartrand TL, Lakin JL (2013). The antecedents and consequences of human behavioral mimicry. Annu Rev Psychol..

[6] Rendell L, Fogarty L, Hoppitt WJ, Morgan TJ, Webster MM, Laland KN (2011). Cognitive culture: theoretical and empirical insights into social learning strategies. Trends Cogn Sci..

[7] Jones SS (2007). Imitation in infancy: the development of mimicry. Psychol Sci..

[8] Young GS, Rogers SJ, Hutman T, Rozga A, Sigman M, Ozonoff S (2011). Imitation from 12 to 24 months in autism and typical development: a longitudinal Rasch analysis. Dev Psychol..

[9] Vanvuchelen M, Roeyers H, De Weerdt W (2011). Do imitation problems reflect a core characteristic in autism? Evidence from a literature review. Res Autism Spectrum Disord..

[10] Poon KK, Watson LR, Baranek GT, Poe MD (2012). To what extent do joint attention, imitation, and object play behaviors in infancy predict later communication and intellectual functioning in ASD?. J Autism Dev Disord..

[11] Iacoboni M, Dapretto M (2006). The mirror neuron system and the consequences of its dysfunction. Nat Rev Neurosci..

[12] Keysers C, Gazzola V (2010). Social neuroscience: mirror neurons recorded in humans. Curr Biol..

[13] Tramacere A, Pievani T, Ferrari PF (2017). Mirror neurons in the tree of life: mosaic evolution, plasticity and exaptation of sensorimotor matching responses. Biol Rev..

[14] Molenberghs P, Cunnington R, Mattingley JB (2012). Brain regions with mirror properties: a meta-analysis of 125 human fMRI studies. Neurosci Biobehav Rev..

[15] Mukamel R, Ekstrom AD, Kaplan J, Iacoboni M, Fried I (2010). Single-neuron responses in humans during execution and observation of actions. Curr Biol..

[16] Rizzolatti G, Sinigaglia C (2016). The mirror mechanism: a basic principle of brain function. Nat Rev Neurosci..

[17] Grill-Spector K, Henson R, Martin A (2006). Repetition and the brain: neural models of stimulus-specific effects. Trends Cogn Sci..

[18] Kilner JM, Neal A, Weiskopf N, Friston KJ, Frith CD (2009). Evidence of mirror neurons in human inferior frontal gyrus. J Neurosci..

[19] Chong TT-J, Cunnington R, Williams MA, Kanwisher N, Mattingley JB (2008). fMRI adaptation reveals mirror neurons in human inferior parietal cortex. Curr Biol..

[20] Iacoboni M, Woods RP, Brass M, Bekkering H, Mazziotta JC, Rizzolatti G (1999). Cortical mechanisms of human imitation. Science..

[21] Leslie KR, Johnson-Frey SH, Grafton ST (2004). Functional imaging of face and hand imitation: towards a motor theory of empathy. Neuroimage..

[22] Uddin LQ, Kaplan JT, Molnar-Szakacs I, Zaidel E, Iacoboni M (2005). Self-face recognition activates a frontoparietal “mirror” network in the right hemisphere: an event-related fMRI study. Neuroimage..

[23] Fan Y, Duncan NW, de Greck M, Northoff G (2011). Is there a core neural network in empathy? An fMRI based quantitative meta-analysis. Neurosci Biobehav Rev..

[24] Killgore WD, Yurgelun-Todd DA (2004). Activation of the amygdala and anterior cingulate during nonconscious processing of sad versus happy faces. Neuroimage..

[25] Pokorny JJ, Hatt NV, Colombi C, Vivanti G, Rogers SJ, Rivera SM (2015). The action observation system when observing hand actions in autism and typical development. Autism Res..

[26] Buccino G, Binkofski F, Riggio L (2004). The mirror neuron system and action recognition. Brai Lang..

[27] Kim S-Y, Choi U-S, Park S-Y, Oh S-H, Yoon H-W, Koh Y-J (2015). Abnormal activation of the social brain network in children with autism spectrum disorder: an fMRI study. Psychiatry Investig..

[28] Sato W, Toichi M, Uono S, Kochiyama T (2012). Impaired social brain network for processing dynamic facial expressions in autism spectrum disorders. BMC Neurosci..

[29] Shafir T, Taylor SF, Atkinson AP, Langenecker SA, Zubieta J-K (2013). Emotion regulation through execution, observation, and imagery of emotional movements. Brain Cogn..

[30] Perkins TJ, Bittar RG, McGillivray JA, Cox II, Stokes MA (2015). Increased premotor cortex activation in high functioning autism during action observation. J Clin Neurosci..

[31] Yang J, Hofmann J (2016). Action observation and imitation in autism spectrum disorders: an ALE meta-analysis of fMRI studies. Brain Imaging Behav..

[32] Bertone A, Mottron L, Jelenic P, Faubert J (2005). Enhanced and diminished visuo-spatial information processing in autism depends on stimulus complexity. Brain..

[33] Chetcuti L, Hudry K, Grant M, Vivanti G (2019). Object-directed imitation in autism spectrum disorder is differentially influenced by motoric task complexity, but not social contextual cues. Autism..

[34] Lange N, Travers BG, Bigler ED, Prigge MB, Froehlich AL, Nielsen JA (2015). Longitudinal volumetric brain changes in autism spectrum disorder ages 6–35 years. Autism Res..

[35] Radua J, Mataix-Cols D, Phillips ML, El-Hage W, Kronhaus D, Cardoner N, et al. A new meta-analytic method for neuroimaging studies that combines reported peak coordinates and statistical parametric maps. Eur Psychiatry. 2012;27(8):605–11. 10.1016/j.eurpsy.2011.04.001 21658917

[36] Moher D, Liberati A, Tetzlaff J, Altman DG, Group P (2009). Preferred reporting items for systematic reviews and meta-analyses: the PRISMA statement. PLoS Med..

[37] Caspers S, Zilles K, Laird AR, Eickhoff SB (2010). ALE meta-analysis of action observation and imitation in the human brain. Neuroimage..

[38] Rizzolatti G, Cattaneo L, Fabbri-Destro M, Rozzi S (2014). Cortical mechanisms underlying the organization of goal-directed actions and mirror neuron-based action understanding. Physiological Rev..

[39] Cracco E, Bardi L, Desmet C, Genschow O, Rigoni D, De Coster L (2018). Automatic imitation: a meta-analysis. Psychol Bull..

[40] Müller VI, Cieslik EC, Laird AR, Fox PT, Radua J, Mataix-Cols D (2018). Ten simple rules for neuroimaging meta-analysis. Neurosci Biobehav Rev..

[41] Jack A, Morris JP (2014). Neocerebellar contributions to social perception in adolescents with autism spectrum disorder. Dev Cogn Neurosci..

[42] Okamoto Y, Kitada R, Tanabe HC, Hayashi MJ, Kochiyama T, Munesue T (2014). Attenuation of the contingency detection effect in the extrastriate body area in autism spectrum disorder. Neurosci Res..

[43] Poulin-Lord M-P, Barbeau EB, Soulières I, Monchi O, Doyon J, Benali H (2014). Increased topographical variability of task-related activation in perceptive and motor associative regions in adult autistics. Neuroimage Clin..

[44] Villalobos ME, Mizuno A, Dahl BC, Kemmotsu N, Müller R-A (2005). Reduced functional connectivity between V1 and inferior frontal cortex associated with visuomotor performance in autism. Neuroimage..

[45] Wadsworth HM, Maximo JO, Lemelman AR, Clayton K, Sivaraman S, Deshpande HD (2017). The action imitation network and motor imitation in children and adolescents with autism. Neuroscience..

[46] Williams JH, Waiter GD, Gilchrist A, Perrett DI, Murray AD, Whiten A (2006). Neural mechanisms of imitation and ‘mirror neuron’ functioning in autistic spectrum disorder. Neuropsychologia..

[47] De Stefani E, De Marco D (2019). Gesture, language and emotional communication: an embodied view of social interaction. Front Psychol..

[48] Lee E, Kang JI, Park IH, Kim J-J, An SK (2008). Is a neutral face really evaluated as being emotionally neutral?. Psychiatry Res..

[49] Leppänen JM, Milders M, Bell JS, Terriere E, Hietanen JK (2004). Depression biases the recognition of emotionally neutral faces. Psychiatry Res..

[50] Kenworthy L, Case L, Harms MB, Martin A, Wallace GL (2010). Adaptive behavior ratings correlate with symptomatology and IQ among individuals with high-functioning autism spectrum disorders. J Autism Dev Disord..

[51] Lai M-C, Lombardo MV, Pasco G, Ruigrok AN, Wheelwright SJ, Sadek SA, et al. A behavioral comparison of male and female adults with high functioning autism spectrum conditions. PloS One. 2011;6(6):e20835. 10.1371/journal.pone.0020835 PMC3113855 21695147

[52] Radua J, Via E, Catani M, Mataix-Cols D (2011). Voxel-based meta-analysis of regional white-matter volume differences in autism spectrum disorder versus healthy controls. Psychol Med..

[53] Radua J, Grau M, Van Den Heuvel OA, De Schotten MT, Stein DJ, Canales-Rodríguez EJ (2014). Multimodal voxel-based meta-analysis of white matter abnormalities in obsessive–compulsive disorder. Neuropsychopharmacology..

[54] Higgins JP, Savović J, Page MJ, Elbers RG, Sterne JA. Assessing risk of bias in a randomized trial, Cochrane Handbook for Systematic Reviews of. Interventions. 2019:205–28.

[55] Borenstein M, Hedges LV, Higgins JP, Rothstein HR (2011). Introduction to meta-analysis.

[56] Egger M, Smith GD, Schneider M, Minder C (1997). Bias in meta-analysis detected by a simple, graphical test. BMJ..

[57] Sterne JA, Sutton AJ, Ioannidis JP, Terrin N, Jones DR, Lau J (2011). Recommendations for examining and interpreting funnel plot asymmetry in meta-analyses of randomised controlled trials. BMJ..

[58] Schwarzer G, Carpenter JR, Rücker G. Meta-analysis with R: Springer; 2015.

[59] Cole EJ, Barraclough NE, Andrews TJ (2019). Reduced connectivity between mentalizing and mirror systems in autism spectrum condition. Neuropsychologia..

[60] Freitag CM, Konrad C, Häberlen M, Kleser C, von Gontard A, Reith W (2008). Perception of biological motion in autism spectrum disorders. Neuropsychologia..

[61] Grèzes J, Wicker B, Berthoz S, De Gelder B (2009). A failure to grasp the affective meaning of actions in autism spectrum disorder subjects. Neuropsychologia..

[62] Hubbard AL, McNealy K, Scott-Van Zeeland AA, Callan DE, Bookheimer SY, Dapretto M (2012). Altered integration of speech and gesture in children with autism spectrum disorders. Brain Behav..

[63] Libero LE, Maximo JO, Deshpande HD, Klinger LG, Klinger MR, Kana RK (2014). The role of mirroring and mentalizing networks in mediating action intentions in autism. Mol Autism..

[64] Marsh LE, AFdC H (2011). Dissociation of mirroring and mentalising systems in autism. Neuroimage..

[65] Wadsworth HM, Maximo JO, Donnelly RJ, Kana RK (2018). Action simulation and mirroring in children with autism spectrum disorders. Behav Brain Res..

[66] Martineau J, Andersson F, Barthélémy C, Cottier J-P, Destrieux C (2010). Atypical activation of the mirror neuron system during perception of hand motion in autism. Brain Res..

[67] McKay LS, Simmons DR, McAleer P, Marjoram D, Piggot J, Pollick FE (2012). Do distinct atypical cortical networks process biological motion information in adults with Autism Spectrum Disorders?. NeuroImage.

[68] Bastiaansen JA, Thioux M, Nanetti L, van der Gaag C, Ketelaars C, Minderaa R, et al. Age-related increase in inferior frontal gyrus activity and social functioning in autism spectrum disorder. Biol Psychiatry. 2011;69(9):832–8. 10.1016/j.biopsych.2010.11.007 21310395

[69] Bookheimer SY, Wang AT, Scott A, Sigman M, Dapretto M (2008). Frontal contributions to face processing differences in autism: evidence from fMRI of inverted face processing. J Int Neuropsychol Soc..

[70] Critchley HD, Daly EM, Bullmore ET, Williams SC, Van Amelsvoort T, Robertson DM (2000). The functional neuroanatomy of social behaviour: changes in cerebral blood flow when people with autistic disorder process facial expressions. Brain..

[71] Dalton KM, Nacewicz BM, Johnstone T, Schaefer HS, Gernsbacher MA, Goldsmith HH (2005). Gaze fixation and the neural circuitry of face processing in autism. Nat Neurosci..

[72] Dapretto M, Davies MS, Pfeifer JH, Scott AA, Sigman M, Bookheimer SY (2006). Understanding emotions in others: mirror neuron dysfunction in children with autism spectrum disorders. Nat Neurosci..

[73] Davies MS, Dapretto M, Sigman M, Sepeta L, Bookheimer SY (2011). Neural bases of gaze and emotion processing in children with autism spectrum disorders. Brain Behav..

[74] Deeley Q, Daly EM, Surguladze S, Page L, Toal F, Robertson D (2007). An event related functional magnetic resonance imaging study of facial emotion processing in Asperger syndrome. Biol Psychiatry..

[75] Greimel E, Schulte-Rüther M, Fink GR, Piefke M, Herpertz-Dahlmann B, Konrad K (2010). Development of neural correlates of empathy from childhood to early adulthood: an fMRI study in boys and adult men. J Neural Transm..

[76] Schneider K, Regenbogen C, Pauly KD, Gossen A, Schneider DA, Mevissen L (2013). Evidence for gender-specific endophenotypes in high-functioning autism spectrum disorder during empathy. Autism Res..

[77] Schulte-Rüther M, Greimel E, Markowitsch HJ, Kamp-Becker I, Remschmidt H, Fink GR (2011). Dysfunctions in brain networks supporting empathy: an fMRI study in adults with autism spectrum disorders. Soc Neurosci..

[78] Carter EJ, Williams DL, Minshew NJ, Lehman JF (2012). Is he being bad? Social and language brain networks during social judgment in children with autism. PLoS One..

[79] Friston KJ (2011). Functional and effective connectivity: a review. Brain Connect..

[80] Kana RK, Uddin LQ, Kenet T, Chugani D, Müller R-A (2014). Brain connectivity in autism. Front Human Neurosci..

[81] Han YM, Chan AS (2017). Disordered cortical connectivity underlies the executive function deficits in children with autism spectrum disorders. Res Dev Disabil..

[82] O’Reilly C, Lewis JD, Elsabbagh M (2017). Is functional brain connectivity atypical in autism? A systematic review of EEG and MEG studies. PLoS One..

[83] Ramos TC, Balardin JB, Sato JR, Fujita A (2018). Abnormal cortico-cerebellar functional connectivity in autism spectrum disorder. Front Syst Neurosci..

[84] Rudie JD, Shehzad Z, Hernandez LM, Colich NL, Bookheimer SY, Iacoboni M (2011). Reduced functional integration and segregation of distributed neural systems underlying social and emotional information processing in autism spectrum disorders. Cerebral Cortex..

[85] Odriozola P, Dajani DR, Burrows CA, Gabard-Durnam LJ, Goodman E, Baez AC (2019). Atypical frontoamygdala functional connectivity in youth with autism. Dev cogn Neurosci..

[86] Festini SB, Zahodne L, Reuter-Lorenz PA (2018). Theoretical perspectives on age differences in brain activation: HAROLD, PASA, CRUNCH—How Do They STAC up? Oxford Research Encyclopedia of Psychology.

[87] Peterson D, Mahajan R, Crocetti D, Mejia A, Mostofsky S (2015). Left-hemispheric microstructural abnormalities in children with high-functioning autism spectrum disorder. Autism Res..

[88] Samartsidis P, Montagna S, Nichols TE, Johnson TD (2017). The coordinate-based meta-analysis of neuroimaging data. Stat Sci..

[89] Philip R, Whalley H, Stanfield A, Sprengelmeyer R, Santos I, Young A (2010). Deficits in facial, body movement and vocal emotional processing in autism spectrum disorders. Psychol Med..

[90] Vanderwal T, Hunyadi E, Grupe DW, Connors CM, Schultz RT (2008). Self, mother and abstract other: an fMRI study of reflective social processing. Neuroimage..

[91] K-i T (2015). Inferior frontal gyrus activation underlies the perception of emotions, while precuneus activation underlies the feeling of emotions during music listening. Behav Neurol..

[92] Kida I, Hoshi Y (2016). Right ventrolateral prefrontal cortex involvement in the integration of emotional processing: Parametric mediation analysis of fMRI. Neuroscience letters..

[93] Hartwigsen G, Neef NE, Camilleri JA, Margulies DS, Eickhoff SB (2018). Functional segregation of the right inferior frontal gyrus: Evidence from coactivation-based parcellation. Cerebral Cortex..

[94] Morawetz C, Bode S, Baudewig J, Kirilina E, Heekeren HR (2016). Changes in effective connectivity between dorsal and ventral prefrontal regions moderate emotion regulation. Cerebral Cortex..

[95] Dougherty CC, Evans DW, Katuwal GJ, Michael AM. Asymmetry of fusiform structure in autism spectrum disorder: trajectory and association with symptom severity. Mol Autism. 2016;7(1):28. 10.1186/s13229-016-0089-5 PMC4879740 27226895

[96] Nielsen JA, Zielinski BA, Ferguson MA, Lainhart JE, Anderson JS. An evaluation of the left-brain vs. right-brain hypothesis with resting state functional connectivity magnetic resonance imaging. PloS one. 2013;8(8):e71275. 10.1371/journal.pone.0071275 PMC3743825 23967180

